# Особенности истинной гинекомастии у взрослых мужчин

**DOI:** 10.14341/probl13491

**Published:** 2024-09-16

**Authors:** С. Х. Эристави, Р. В. Роживанов, Л. В. Никанкина, Г. С. Колесникова, Е. Р. Роживанова, Е. Н. Андреева, Г. А. Мельниченко, Н. Г. Мокрышева

**Affiliations:** Национальный медицинский исследовательский центр эндокринологии; Национальный медицинский исследовательский центр эндокринологии; Национальный медицинский исследовательский центр эндокринологии; Национальный медицинский исследовательский центр эндокринологии; Национальный медицинский исследовательский центр эндокринологии; Национальный медицинский исследовательский центр эндокринологии; Российский университет медицины; Национальный медицинский исследовательский центр эндокринологии; Национальный медицинский исследовательский центр эндокринологии

**Keywords:** Гинекомастия, мужчины, взрослые

## Abstract

**ОБОСНОВАНИЕ:**

ОБОСНОВАНИЕ. За последние годы существенно возросла обращаемость взрослых мужчин по поводу гинекомастии. Вызывает интерес изучение особенностей заболевания у этих пациентов.

**ЦЕЛЬ:**

ЦЕЛЬ. Установить основные характеристики остро возникшей гинекомастии у взрослых мужчин.

**МАТЕРИАЛЫ И МЕТОДЫ:**

МАТЕРИАЛЫ И МЕТОДЫ. Сплошное одномоментное исследование, включившее 160 взрослых мужчин с остро возникшей гинекомастией, обратившихся в ГНЦ ФГБУ НМИЦ Эндокринологии Минздрава РФ. У всех пациентов оценивали: состояние грудных желез, общий билирубин, печеночные трансаминазы, креатинин, мочевину, лютеинизирующий гормон (ЛГ), пролактин, глобулин, связывающий половые гормоны (ГСПГ), эстрадиол, общий тестостерон, альфафетопротеин (АФП), хорионический гонадотропин (ХГ). Базовый уровень статистической значимости p<0,05.

**РЕЗУЛЬТАТЫ:**

РЕЗУЛЬТАТЫ. Обращаемость с гинекомастией увеличилась от 5,4% в 2020 г. до 14,4% в 2024 г. Опухолевые формы гинекомастии диагностировались редко: 1,2% (95% ДИ 0,0; 3,0) случаев. У 30% (95% ДИ 22,9; 37,1) мужчин гинекомастия была обусловлена приемом анаболических стероидов в целях атлетической стимуляции, у 11,2% (95% ДИ 6,4; 16,1) была гепатогенной, у 7,5% (95% ДИ 3,4; 11,6) — обусловленной повышением ГСПГ. 47,5% (95% ДИ 39,8; 55,2) составила эндокринная неопухолевая форма гинекомастии, обусловленная избытком массы тела с формированием изменений уровней половых гормонов. Для пациентов, принимавших анаболические стероиды, был характерен молодой возраст, а также снижение уровня ЛГ и повышение уровня тестостерона. Группа пациентов с повышенным ГСПГ не имела клинически значимых особенностей. Для мужчин из группы гепатогенной гинекомастии была характерна гиперэст­рогения. Для пациентов из группы с изменениями уровней половых гормонов был характерен высокий индекс массы тела (ИМТ), а также либо повышение уровня эстрадиола, либо снижение уровня тестостерона или их сочетание.

**ЗАКЛЮЧЕНИЕ:**

ЗАКЛЮЧЕНИЕ. Число обращений взрослых мужчин с остро возникшей гинекомастией прогрессивно увеличивается. В обследованной выборке пациентов основными причинами гинекомастии являлись прием пациентами анаболических стероидов, нарушения функции печени и увеличение массы тела с формированием изменений уровней половых гормонов. Для пациентов, принимавших анаболические стероиды, было характерно медикаментозно обусловленное повышение уровней тестостерона и эстрадиола, что сопровождалось подавлением гонадотропной функции гипофиза. Повышение эстрадиола также было характерно для пациентов с гепатогенной формой гинекомастии и мужчин с избытком массы тела с формированием изменений уровней половых гормонов.

## Abstract (en)

**BACKGROUND:**

BACKGROUND: In recent years, the incidence of gynecomastia in adult men has increased significantly. It is of interest to study the specific features of the disease in these patients.

**AIM:**

AIM: To identify the main characteristics of acute gynecomastia in adult men.

**MATERIALS AND METHODS:**

MATERIALS AND METHODS: A continuous one-stage study including 160 adult males with acute onset gynecomastia, who were he was treated in Endocrinology Research Centre, Moscow. Total bilirubin, hepatic transaminases, creatinine, urea, luteinizing hormone, prolactin, sex hormone binding globulin, estradiol, total testosterone, alpha-fetoprotein, chorionic gonadotropin and mammary gland condition were evaluated in all patients. Baseline significance threshold level of p<0.05.

**RESULTS:**

RESULTS: The incidence of gynecomastia increased from 5,4% in 2020 to 14,4% in 2024. Tumor forms of gynecomastia were rare, with 1,2% (95% CI 0,0; 3,0) of cases. In 30% (95% CI 22,9; 37,1) of men, gynecomastia was due to the intake of anabolic steroids for athletic stimulation. In 11,2% (95% CI 6,4; 16,1) of patients, gynecomastia was hepatogenic. In 7,5% (95% CI 3,4; 11,6), it was due to elevated sex hormone binding globulin. 47,5% (95% CI 39,8; 55,2) were endocrine non-tumorigenic form of gynecomastia due to excess body weight with formation of changes in sex hormone levels. The patients who took anabolic steroids were characterized by young age, as well as decreased luteinizing hormone levels and increased testosterone levels. The group of patients with elevated sex hormone binding globulin had no clinically significant features. Men from the group of hepatogenic gynecomastia were characterized by hyperestrogenism. Patients in the group with altered sex hormone levels were characterized by high body mass index and either increased estradiol or decreased testosterone or a combination of both.

**CONCLUSION:**

CONCLUSION: The number of adult male patients with acute gynecomastia is progressively increasing. In the examined sample of patients, the main causes of gynecomastia were patients taking anabolic steroids, liver dysfunction and weight gain with the formation of changes in sex hormone levels. Patients taking anabolic steroids were characterized by a drug-­induced increase in testosterone and estradiol levels, which was accompanied by suppression of pituitary gonadotropic function. Estradiol elevation was also characteristic of patients with hepatogenic form of gynecomastia and men with excess body weight with formation of changes in sex hormone levels.

## ОБОСНОВАНИЕ

За последние годы существенно возросла обращаемость взрослых мужчин по поводу гинекомастии. Гинекомастия — доброкачественное увеличение грудных желез у мужчин, которое может быть физиологическим, патологическим или идиопатическим [1]. Физиологическая гинекомастия может развиться в период полового созревания, и впоследствии пациентов как правило не беспокоит. Патологическая гинекомастия связана с различными эндокринными, генетическими нарушениями, системными заболеваниями, паранеопластическими процессами, или может быть ятрогенной [2]. В ряде случаев патологическая гинекомастия не связана с генетическими нарушениями и возникает остро у взрослых мужчин. Для таких форм гинекомастии чаще всего характерно нарушение баланса между эстрогенами и андрогенами или прямое воздействие лекарственного препарата на грудную железу [3][4]. Учитывая возросшую обращаемость мужчин с соответствующими жалобами, а также отсутствие в литературе данных о характеристиках форм гинекомастии, не связанных с препубертатной и пубертатной патологиями, мы запланировали исследование для изучения особенностей остро возникших форм истинной гинекомастии у взрослых мужчин, не имеющих отягощений препубертатного и пубертатного анамнеза.

## ЦЕЛЬ

Установить основные характеристики остро возникшей гинекомастии у взрослых мужчин.

## МАТЕРИАЛЫ И МЕТОДЫ

## Место и время проведения исследования

ФГБУ НМИЦ Эндокринологии Министерства здравоохранения РФ, Москва. Исследование выполнено в период с апреля по июль 2024 г.

## Изучаемые популяции

Формирование групп проводилось из пациентов, обратившихся за медицинской помощью в ФГБУ НМИЦ Эндокринологии Министерства здравоохранения РФ.

Критерии включения: возраст старше 25 лет, мужской пол, остро возникшая истинная гинекомастия.

Критерии невключения: препубертатная или пубертатная гинекомастия, недееспособность.

Критерии исключения: отказ от выполнения программы исследования (все проводимые исследования являлись рутинными, однако у пациентов было право отказаться от них полностью или частично, и такие пациенты в исследование не включались).

## Способ формирования выборки из изучаемой популяции

Выборка формировалась сплошным способом.

## Дизайн исследования

Сплошное одномоментное исследование взрослых мужчин с остро возникшей истинной гинекомастией.

## Методы

Физикальное обследование включало общий осмотр с определением характеристик оволосения, в том числе лобкового, типа телосложения, состояния грудных желез, а также оценку состояния наружных половых органов. У всех пациентов регистрировались следующие результаты обследования: общий билирубин, печеночные трансаминазы, креатинин, мочевина, лютеинизирующий гормон (ЛГ), пролактин (а также биологически активный пролактин при выявлении гиперпролактинемии), глобулин, связывающий половые гормоны (ГСПГ), эстрадиол, общий тестостерон, альфафетопротеин (АФП), хорионический гонадотропин (ХГ). Уровни общего тестостерона (референсный интервал (РИ) 12,0–28,2 нмоль/л), ЛГ (РИ 2,5–11,0 ЕД/л), эстрадиола (РИ 19,7–242,0 пмоль/л), пролактина (РИ 60–355, мЕд/л), ХГЧ (РИ менее 2,0 нг/мл) определялись методом иммунохемилюминесцентного анализа (ИХЛА) на автоматическом анализаторе Vitros ECi 3600 (Ortho-Clinical Diagnostics), ГСПГ (РИ 20,6–76,7 нмоль/л) на анализаторе Cobas 6000 (Roche) и АФП (РИ 0–6,4 МЕ/мл) на анализаторе Architect 2000 (Abbott). Концентрацию биохимических показателей сыворотки крови — аланинаминотрансферазы (АЛТ) (РИ 0–55 Ед/л), аспартатаминотрансферазы (АСТ) (РИ 5–34 Ед/л), общего билирубина (РИ 3,4–20,5 мкмоль/л), креатинина (РИ 63–110 мкмоль/л), мочевины (РИ 3,5–7,2 ммоль/л) определяли на биохимическом анализаторе Architect 8000 (Abbott). Ультразвуковое исследование грудных желез выполнялось на аппарате Pro Focus 2202 (B-K Medical ApS, Denmark).

## Описание медицинского вмешательства

Всем пациентам осуществлялся забор крови в пробирки типа «вакутейнер» в утреннее время натощак из локтевой вены.

## Статистический анализ

Принципы расчета размера выборки

Размер выборки предварительно не рассчитывался.

Методы статистического анализа данных

Статистическая обработка полученных данных была проведена с использованием пакета прикладных программ STATISTICA (Stat Soft Inc., США, версия 10.0). Сравнение по качественным признакам осуществлялось непараметрическим методом χ² с поправкой Йетса, по количественным — U тестом Манна-Уитни. Базовый пороговый уровень значимости p<0,05. При множественных сравнениях применялась поправка Бонферрони. Результаты исследований представлены в виде медиан параметров, интерквартильного отрезка для количественных признаков, а также абсолютных чисел и процентов для качественных признаков.

## Этическая экспертиза

Проведение исследования одобрено Локальным этическим комитетом ФГБУ «НМИЦ Эндокринологии» Минздрава России (протокол №10 от 22.05.2024).

## РЕЗУЛЬТАТЫ

С 2020 по 2024 гг. обращаемость мужчин в ФГБУ НМИЦ Эндокринологии Минздрава РФ с жалобами на остро развившуюся истинную гинекомастию прогрессивно увеличивается, составляя 5,4% (20 из 371 впервые обратившихся пациентов) в 2020 г., 4,9% (23 из 468) в 2021 г., 6,5% (35 из 572) в 2022 г., 7,3% (38 из 522) в 2023 г., и 14,4% (42 из 292) в 2024 г., различия являются статистически значимыми (р<0,001 по сравнению 2024 г. и р=0,002 по сравнению с 2023 г., χ² с поправкой Йетса, применялась поправка Бонферрони, уровень статистической значимости р<0,0125). Характеристики выборки пациентов представлены в табл. 1.

**Table:** Таблица 1. Характеристики выборки (n=160) Примечание: Me [ 25%; 75%].

Показатель	Значение
Возраст, лет	32 [ 29; 37]
ИМТ, кг/м²	27,3 [ 24,8; 28,9]
Степень гинекомастии справа	2 [ 2; 3]
Степень гинекомастии слева	2 [ 2; 3]
Длительность с дебюта, нед.	11 [ 9; 14]
Общий билирубин, мкмоль/л	13,9 [ 10,4; 17,1]
АСТ, ЕД/мл	27 [ 22; 30]
АЛТ, ЕД/мл	27 [ 19; 38]
Креатинин, мкмоль/л	6 [ 69; 84]
Мочевина, ммоль/л	4,9 [ 4,3; 5,8]
ЛГ, ЕД/л	3,3 [ 1,7; 4;4]
Пролактин, мЕд/л	289 [ 185,5; 340]
ГСПГ, нмоль/л	33,5 [ 24,9; 51,2]
Эстрадиол, пмоль/л	105,2 [ 75,6; 281,2]
Общий тестостерон, нмоль/л	16,3 [ 12,2; 37]
АФП, МЕ/мл	1,4 [ 1; 2,1]
ХГ, нмоль/л	0 [ 0; 0]

У пациентов обследованной выборки не было выявлено почечной недостаточности, и у никого не отмечалось повышенного уровня АФП. У одного из пациентов 27 лет (0,6% (95%ДИ 0,0; 1,8) случаев) гинекомастия была обусловлена наличием семиномы с гиперпродукцией ХГ. Кроме повышения ХГ, у него были выявлены гиперандрогения и гиперэстрогения. Еще у одного пациента 38 лет гинекомастия была обусловлена гиперпролактинемией вследствие пролактиномы (0,6% (95% ДИ 0,0; 1,8) случаев) с гипогонадизмом. Кроме того, гиперпролактинемия обусловила еще 2 случая гинекомастии (1,2% (95% ДИ 0,0; 3,0)), однако сама она являлась ятрогенной и была связана с приемом амитриптилина в одном случае и пароксетина в другом. У этих пациентов гиперпролактинемия сопровождалась нормальным уровнем тестостерона и эстрадиола. Еще в двух случаях медикаментозной гинекомастии (1,2% (95% ДИ 0,0;3,0)) она была вызвана приемом дигоксина и нифедипина. У этих пациентов были выявлены нормальные показатели тестостерона и эстрадиола. Самой многочисленной группой (48 мужчин, 30% (95% ДИ 22,9; 37,1)) медикаментозной гинекомастии являлись пациенты, принимавшие разные препараты анаболических стероидов в целях атлетической стимуляции. Результаты обследования представлены в таблице 2. Итого число случаев гинекомастии, обусловленных эндокринно-активными опухолями, составило 2 случая (1,2% (95% ДИ 0,0; 3,0), а медикаментозными причинами — 52 случая (32,5% (95% ДИ 25,2; 39,8)).

**Table:** Таблица 2. Результаты обследования мужчин в зависимости от причин гинекомастии Примечание: Me [ 25%; 75%], U тест Манна-Уитни, проводились множественные сравнения, применялась поправка Бонферрони, уровень статистической значимости p<0,00064.

Показатель	1. Прием анаболических стероидов, n=48	2. Повышение уровня ГСПГ, n=12	3. Гепатопатия, n=18	4. Изменения половых гормонов, n=76	p 1–2	p 1–3	p 1–4	p 2–3	p 2–4	p 3–4
Возраст, лет	28[ 27; 30]	39[ 37; 42]	37[ 31; 44]	33[ 31; 37]	0,0000	0,0000	0,0000	0,539	0,003	0,098
ИМТ, кг/м²	24,8[ 23,6; 27,4]	27,5[ 26,2; 28,0]	24,4[ 23,5; 24,8]	28,7[ 27,5; 30,5]	0,011	0,052	0,0000	0,0002	0,002	0,0000
Степень гинекомастии справа	2[ 2; 3]	2[ 2; 2]	2[ 2; 2]	2[ 2; 3]	0,624	0,767	0,807	0,815	0,692	0,889
Степень гинекомастии слева	2[ 2; 2]	2[ 2; 2]	2[ 2; 2]	2[ 2; 3]	0,691	0,449	0,179	0,815	0,605	0,762
Длительность с дебюта, нед.	9[ 8; 12]	12[ 9; 14]	11[ 9; 12]	13[ 10; 15]	0,202	0,121	0,0000	0,750	0,165	0,034
Общий билирубин, мкмоль/л	15,4[ 13,5; 17,2]	8,6[ 7,8; 12,7]	25,5[ 18,1; 28,6]	12[ 8,4; 16,1]	0,001	0,0000	0,0000	0,0000	0,255	0,0000
АСТ, ЕД/мл	27[ 24; 47]	18[ 14; 26]	45[ 37; 115]	25[ 21; 28]	0,0001	0,001	0,001	0,0000	0,007	0,0000
АЛТ, ЕД/мл	26[ 18; 47]	24[ 19; 38]	45[ 25; 98]	27[ 18; 32]	0,963	0,012	0,799	0,049	0,883	0,007
ЛГ, ЕД/л	1,1[ 0,4; 1,5]	3,8[ 3,1; 4,6]	4,1[ 3,5; 4,9]	4,1[ 3,1; 4,8]	0,0000	0,0000	0,0000	0,471	0,580	0,751
Пролактин, мЕд/л	320[ 220; 384]	301[ 287; 336]	314[ 296; 342]	187[ 145; 294]	0,598	0,936	0,0000	0,421	0,002	0,0000
ГСПГ, нмоль/л	40,5[ 27,9; 72,7]	89,2[ 83,2; 95,2]	37,7[ 25,2; 48,1]	28,3[ 22,7; 37,7]	0,0000	0,476	0,0002	0,0000	0,0000	0,066
Эстрадиол, пмоль/л	94,1[ 75,5; 276,6]	38,1[ 32,7; 45,2]	274,3[ 96,1; 286,4]	157,4[ 94,9; 293,8]	0,0000	0,119	0,015	0,0001	0,0000	0,977
Общий тестостерон, нмоль/л	38,4[ 37,5; 40,6]	13,4[ 12,5; 18,3]	16,5[ 11,3; 19,0]	12,9[ 10,7; 15,0]	0,0000	0,0000	0,0000	1,000	0,099	0,080

У 18 мужчин (11,2% (95% ДИ 6,4; 16,1)) гинекомастия являлась гепатогенной. У двух из них в анамнезе присутствовали токсические повреждения печени, еще у четырех — гепатит, у 8 — синдром Жильбера и у четырех особенностей анамнеза выявлено не было, но их гинекомастия с уверенностью может считаться гепатогенной, так как отмечались либо повышение билирубина, либо печеночных трансаминаз, либо их сочетание, а также повышение эстрадиола (табл. 2).

У 12 пациентов (7,5% (95% ДИ 3,4; 11,6)) гинекомастия была обусловлена повышением ГСПГ (табл. 2). Особенностей анамнеза пациентов с повышенным ГСПГ установлено не было. Так как у этих пациентов не было иных причин для развития гинекомастии, кроме повышения ГСПГ, а ГСПГ вырабатывается печенью, то, по нашему мнению, такую гинекомастию следует считать гепатогенной. Итого в нашей выборке представлено 30 случаев гепатогенной гинекомастии (18,7% (95% ДИ 12,7; 24,8)).

Оставшиеся 76 случаев (47,5% (95% ДИ 39,8; 55,2)) составила эндокринная неопухолевая форма гинекомастии, обусловленная избытком массы тела с формированием изменений уровней половых гормонов (табл. 2).

При сравнении групп пациентов с гинекомастией, обусловленной разными причинами, было установлено, что для мужчин, принимавших анаболические стероиды, был характерен статистически значимо меньший возраст, а также снижение уровня ЛГ и повышение уровня тестостерона. Иные выявленные статистически значимые различия этой группы с другими не являлись клинически значимыми, так как исследуемые уровни показателей соответствовали нормальным значениям, за исключением эстрадиола, который был статистически значимо выше по сравнению с группой повышенного ГСПГ. Группа пациентов с повышенным ГСПГ не имела клинически значимых особенностей, за исключением самого уровня ГСПГ, но он и являлся критерием деления на группы. Для мужчин из группы гепатогенной гинекомастии было характерно статистически значимое повышение уровня билирубина и печеночных трансаминаз, что логично, так как эти признаки являлись критерием деления на группы. Кроме этого, для этой группы был характерен статистически значимо более высокий уровень эстрадиола по сравнению с мужчинами из группы с повышенным ГСПГ. Для пациентов из группы с изменениями уровней половых гормонов был характерен статистически значимо более высокий ИМТ по сравнению с мужчинами, принимавшими анаболические стероиды и пациентами с гепатогенной гинекомастией, а также статистически значимо более высокий уровень эстрадиола по сравнению с мужчинами из группы с повышенным ГСПГ.

## ОБСУЖДЕНИЕ

## Репрезентативность выборок

Учитывая, что выборка пациентов была сформирована исключительно в ФГБУ НМИЦ Эндокринологии, распространенность и структура гинекомастии в других выборках может отличаться.

## Сопоставление с другими публикациями

Известно, что ведущую роль в развитии грудных желез занимают половые гормоны и пролактин. Поскольку у мужчин существует баланс между эстрогенами и андрогенами, любое патологическое состояние или прием лекарств, которые увеличивают уровень циркулирующих эстрогенов или уменьшают уровень циркулирующих андрогенов, может вызвать гинекомастию. Хотя пролактин не имеет ведущей роли в развитии гинекомастии и она редко выявляется при гиперпролактинемии, тем не менее он может стимулировать пролиферацию эпителиальных клеток грудной железы у мужчин при наличии локальной высокой чувствительности к эстрогенам [5]. Это подтверждают результаты нашего исследования, продемонстрировавшего всего три случая гиперпролактинемии: два — на фоне приема антидепрессантов и один — вследствие пролактиномы, сочетанной с дефицитом тестостерона. В некоторых работах авторы описывают роль рецепторов ХГ в тканях грудной железы у мужчин в развитии гинекомастии [6]. Возможно, этим фактом была обусловлена гинекомастия у мужчины с семиномой, но все-таки более вероятно, что в случае нашего пациента гинекомастия развилась не вследствие прямого действия ХГ на грудную железу, а опосредованного его действия через гиперандрогению, а далее гиперэстрогению [7].

Установлено, что около 20% гинекомастии вызвано лекарствами или экзогенными химическими веществами [8]. В нашей работе гинекомастия была обусловлена медикаментозными причинами у 32,5% (95% ДИ 25,2; 39,8) мужчин. Например, один из наших пациентов получал терапию дигоксином, в отношении которого известно, что он способен связываться с рецепторами эстрогена [9]. Но наиболее многочисленной группой медикаментозной гинекомастии были мужчины, принимавшие анаболические стероиды. Эти результаты сопоставимы с данными других исследователей. Появление гинекомастии широко описано у бодибилдеров и спортсменов после введения ароматизирующихся андрогенов (анаболических стероидов) [10]. Применение анаболических стероидов приводит к подавлению выработки ЛГ, что помогает распознавать таких пациентов [11].

Данных по распространенности гепатогенной гинекомастии в литературе не представлено, что связано с разной интерпретацией патогенеза. Так, некоторые авторы не относят гинекомастию к гепатогенной даже при циррозе печени, а объясняют ее патогенез не гепатопатией как таковой, а повышением уровнем ГСПГ, который связывает тестостерон более активно (что приводит к снижению его биологически активной свободной фракции, нарушая андрогенный/эстрогенный баланс), чем эстрадиол, приводя к гинекомастии [12]. Другие авторы подтверждают роль гепатопатии в нарушении метаболизма активации и инактивации эстрогенов в печени [13]. Таким образом, хотя связь гинекомастии с заболеваниями печени очевидна, текущие данные по патогенезу противоречивы, и точный механизм остается неясным.

Отдельный интерес вызывает многочисленная группа пациентов с эндокринной неопухолевой формой гинекомастии, обусловленной избытком массы тела с формированием изменений уровней половых гормонов. Известно, что повышенная выработка лептина при ожирении может стимулировать активность ароматазы в жировой ткани, рост эпителиальных клеток в грудной железе и повышать чувствительность эпителиальных клеток к эстрогену [14]. Повышение активности ароматазы в жировой ткани может быть и не связанным с лептином, а быть генетически обусловленным [15]. Кроме того, как на фоне ожирения, так и без такового, у мужчины может встречаться гипогонадизм, для которого характерно снижение уровня тестостерона и, следовательно, изменение баланса в сторону увеличения действия эстрогенов [16]. Подобные признаки наблюдались у 47,5% (95% ДИ 39,8; 55,2) обследованных нами мужчин. Так как описанные патологические изменения потенциально обратимы [17], эти мужчины могут рассматриваться как кандидаты на медикаментозное лечение.

## Клиническая значимость результатов

Подавление уровня ЛГ может являться маркером, позволяющим выявить воздействие анаболических стероидов у мужчин с высоким андрогенным статусом, которые отрицают медикаментозную атлетическую стимуляцию. Выявление группы мужчин с избытком массы тела и изменениями уровней половых гормонов позволяет рассматривать их как кандидатов на медикаментозную терапию как эндокринных нарушений, так и гинекомастии.

## Ограничения исследования

Ограничениями исследования являются возможные проблемы с репрезентативностью выборки в отношении общей популяции (указаны в соответствующем разделе).

## Направления дальнейших исследований

В продолжение проведенного исследования планируется изучить методы коррекции остро возникшей истинной гинекомастии у взрослых мужчин.

## ЗАКЛЮЧЕНИЕ

Число обращений взрослых мужчин с остро возникшей гинекомастией прогрессивно увеличивается. В обследованной выборке основными причинами гинекомастии являлись прием пациентами анаболических стероидов, нарушения функции печени и увеличение массы тела с формированием изменений уровней половых гормонов. Для пациентов, принимавших анаболические стероиды, было характерно медикаментозно обусловленное повышение уровней тестостерона и эстрадиола, что сопровождалось подавлением гонадотропной функции гипофиза. Повышение эстрадиола также было характерно для пациентов с гепатогенной формой гинекомастии и мужчин с избытком массы тела с формированием изменений уровней половых гормонов.

## ДОПОЛНИТЕЛЬНАЯ ИНФОРМАЦИЯ

Источник финансирования. Работа выполнена по инициативе авторов без привлечения финансирования.

Конфликт интересов. Авторы данной статьи подтвердили отсутствие конфликта интересов, о котором необходимо сообщить.

Выражение признательности. Авторы выражают искреннюю благодарность пациентам, принявшим участие в проведении исследования.

Участие авторов. Все авторы одобрили финальную версию статьи перед публикацией, выразили согласие нести ответственность за все аспекты работы, подразумевающую надлежащее изучение и решение вопросов, связанных с точностью или добросовестностью любой части работы.
